# Prognostic value and clinical net benefit of global longitudinal strain in sepsis: a prospective study with internal bootstrap validation

**DOI:** 10.3389/fcvm.2026.1835581

**Published:** 2026-07-22

**Authors:** Xuan Dai, Shuhao Li, Hanmei Ge, Chenyu Zhang, Chunlin Hu, Haixia Xiong, Hongyan Wei

**Affiliations:** 1 Department of Emergency Medicine, The First Affiliated Hospital of Sun Yat-sen University, Guangzhou, China; 2 Department of Critical Care Medicine, The First Affiliated Hospital of Sun Yat-sen University, Guangzhou, China; 3 Department of Division of Nephrology, The Third Affiliated Hospital of Sun Yat-sen University, Guangzhou, China

**Keywords:** decision curve analysis, emergency critical care, global longitudinal strain, nomogram, sepsis-induced myocardial dysfunction, speckle-tracking echocardiography

## Abstract

**Objective:**

Conventional left ventricular ejection fraction (LVEF) often fails to detect early sepsis-induced myocardial dysfunction (SIMD). This study aimed to evaluate the prognostic utility of speckle-tracking echocardiography (STE)-derived global longitudinal strain (GLS) and to develop an internally validated clinical nomogram with demonstrated net benefit for predicting 28-day mortality in sepsis patients.

**Methods:**

This prospective observational study enrolled 46 patients presenting with sepsis or septic shock at the Emergency Department of The First Affiliated Hospital of Sun Yat-sen University (February–June 2023). STE-derived average GLS (GLS_AVG), conventional echocardiography, and cardiac biomarkers were evaluated within 24 h of diagnosis. Prognostic performance was assessed using restricted multivariable logistic regression, Receiver Operating Characteristic (ROC) curves, and DeLong's test. A predictive nomogram was constructed, validated via 1,000 bootstrap resamples, and evaluated for clinical utility using Decision Curve Analysis (DCA).

**Results:**

The 28-day mortality rate was 23.9% (11/46). Compared to survivors, non-survivors exhibited significantly impaired GLS_AVG (−10.09 ± 4.18% vs. −14.69 ± 3.13%, *P* < 0.001) and higher SOFA scores (11.55 ± 3.11 vs. 7.63 ± 3.15, *P* = 0.001). GLS_AVG strongly correlated with SOFA (*r* = 0.663) and NT-proBNP peak (*r* = 0.424). ROC analysis demonstrated that GLS_AVG (AUC: 0.796) outperformed conventional LVEF (AUC: 0.706) in predicting mortality. The optimal GLS_AVG cutoff was > −11.0% (sensitivity 72.7%, specificity 82.9%). In restricted multivariable analysis, GLS_AVG showed a borderline independent association with 28-day mortality in the restricted multivariable model (OR: 1.266; 95% CI: 0.995–1.676; *P* = 0.067). The formulated GLS-SOFA nomogram showed excellent calibration upon 1,000-bootstrap internal validation. Crucially, DCA confirmed that integrating GLS yielded a substantially higher clinical net benefit across a wide range of threshold probabilities compared to relying on LVEF alone.

**Conclusion:**

STE-derived GLS is a highly sensitive, relatively load-independent marker of early myocardial dysfunction in sepsis, significantly outperforming LVEF in predicting 28-day mortality. The validated GLS-SOFA nomogram and decision curve models provide a practical, high-yield tool to enhance risk stratification and guide early personalized hemodynamic resuscitation in emergency settings.

## 1 Introduction

Sepsis, defined as a life-threatening systemic inflammatory response and organ dysfunction caused by a dysregulated host response to infection, remains a leading cause of morbidity and mortality globally (1). In 2017 alone, an estimated 48.9 million incident cases of sepsis were recorded worldwide, resulting in approximately 11 million sepsis-related deaths, accounting for nearly 20% of all global deaths (2). The progression from sepsis to septic shock is characterized by profound circulatory, cellular, and metabolic abnormalities, drastically increasing the risk of death (1). The initial presentation and the most critical window for early goal-directed resuscitation predominantly occur in the Emergency Department (ED), where rapid and accurate risk stratification is paramount to guiding aggressive hemodynamic support and improving patient outcomes.

A critical but frequently under-recognized complication contributing to this exceptionally high mortality is sepsis-induced myocardial dysfunction (SIMD). First described by Parker et al. in 1984 as a profound yet reversible myocardial depression (3), SIMD is a complex, multi-faceted syndrome. It affects approximately 10%–70% of patients presenting with severe sepsis or septic shock (4, 5). The presence of SIMD significantly worsens the clinical trajectory, with associated mortality rates soaring up to 70% (6). The pathogenesis involves an overwhelming release of pro-inflammatory cytokines, mitochondrial dysfunction, intracellular calcium mishandling, and microvascular thrombosis, collectively leading to impaired cardiac contractility and compliance (4). Autopsy studies have further revealed both macroscopic and microscopic cardiac structural alterations in surgical intensive care patients with sepsis, underscoring the severe physical toll of this condition on the myocardium (7).

Despite its profound clinical significance, the early and precise detection of SIMD remains a major diagnostic challenge in acute settings (8). Conventional echocardiography predominantly relies on the left ventricular ejection fraction (LVEF) to assess systolic function (9). However, LVEF is highly dependent on loading conditions—both preload and afterload—which are drastically altered in the early stages of sepsis due to profound vasodilation, aggressive intravenous fluid resuscitation, and the administration of high-dose vasoactive agents (5). Consequently, LVEF often appears falsely “normal” or hyperdynamic in the hyperkinetic phase of septic shock, masking underlying intrinsic myocardial depression (4). Furthermore, cardiac biomarkers such as Troponin T (TnT) and N-terminal pro-brain natriuretic peptide (NT-proBNP), while helpful, lack specificity for SIMD, and their physiological clearance is frequently confounded by concurrent sepsis-induced multiorgan damage (5, 6).

Speckle tracking imaging (STI), an advanced two-dimensional echocardiographic technique introduced in 2004 (10), has revolutionized the non-invasive assessment of myocardial mechanics. Unlike conventional LVEF, STI provides a highly sensitive, angle-independent, and comprehensive analysis of myocardial deformation (11). Among the parameters derived from STI, global longitudinal strain (GLS) has emerged as an exceptionally robust index (12). Although GLS is less influenced by loading conditions than LVEF, acute hemodynamic fluctuations in sepsis may still partially affect strain values; thus, GLS should be considered a relatively load-independent parameter. The left ventricular subendocardial longitudinal myocardial fibers are highly susceptible to early ischemic injury, increased wall stress, and inflammatory toxins (11). Impairment of these specific subendocardial fibers often precedes global systolic dysfunction. Therefore, GLS can detect subtle, subclinical myocardial dysfunction long before a noticeable drop in LVEF becomes apparent (12).

A growing body of literature has highlighted the prognostic superiority of GLS in critically ill patients. Recent reviews and clinical evaluations have established a strong correlation between reduced (less negative) GLS and elevated short-term mortality in patients with severe sepsis and septic shock, whereas LVEF fails to demonstrate a consistent prognostic association (6, 12). Furthermore, impaired GLS has been tightly linked to severe systemic manifestations of sepsis and indicates a greater severity of multiorgan dysfunction (11, 12). This suggests that GLS reflects not only primary cardiac dysfunction but also the severity of systemic hypoperfusion and impending physiological collapse.

Despite these promising findings, a crucial gap remains in the current diagnostic landscape. The vast majority of sophisticated cardiovascular evaluations in sepsis are conducted within Intensive Care Unit (ICU) settings, often evaluating patients hours or even days after the initial hemodynamic insult. However, the most critical window for early recognition and targeted intervention occurs immediately upon presentation in the ED (8). Evaluating myocardial mechanics at this nascent stage could drastically alter initial resuscitation protocols and tailor fluid or inotropic therapies (8). Additionally, while previous studies have identified GLS as a valuable metric, few have translated this advanced imaging parameter into a comprehensive, internally validated clinical prediction tool that quantifies actual net benefit for frontline emergency physicians.

To address these critical limitations, this prospective observational study aims to evaluate the clinical utility of early STI-derived GLS in predicting 28-day mortality among patients presenting with sepsis or septic shock in the ED. By directly comparing GLS with conventional echocardiographic parameters and integrating it with established clinical severity scores, we seek to construct a robust, internally validated predictive nomogram. Ultimately, through Decision Curve Analysis (DCA), this study intends to demonstrate the tangible clinical net benefit of incorporating advanced strain imaging into the early emergency triage and hemodynamic management of sepsis.

## 2 Materials and methods

### 2.1 Research subjects

This prospective observational study was approved by the Ethics Committee for Clinical Research and Laboratory Animals of the First Affiliated Hospital of Sun Yat-sen University (Ethics Number: [2023]122). The study enrolled patients with sepsis who were initially diagnosed in the Emergency Department of the First Affiliated Hospital of Sun Yat-sen University between February 2023 and June 2023.

The inclusion criteria were as follows: (1) age ≥18 years; (2) meeting the Sepsis 3.0 diagnostic criteria established by the Society of Critical Care Medicine (SCCM) and the European Society of Intensive Care Medicine (ESICM) in 2016. Specifically, patients were required to have a suspected or confirmed infection along with a Sequential Organ Failure Assessment (SOFA) score ≥2. The diagnostic criteria for septic shock included the requirement of vasoactive drugs to maintain a mean arterial pressure (MAP) ≥ 65 mmHg and a blood lactate concentration >2 mmol/L despite adequate fluid resuscitation.

Exclusion criteria comprised acute coronary syndrome, congenital heart disease, acute myocarditis, cardiomyopathy, severe valvular disease, major trauma, post-percutaneous coronary intervention (PCI), history of cardiac surgery, post-cardiopulmonary resuscitation, advanced malignant tumors, age >85 years, and poor ultrasound image quality. Patients were primarily categorized into a survival group and a non-survival group based on their 28-day prognosis. Additionally, for baseline comparative purposes, patients were stratified into a high-strain group (GLS < −14%) and a low-strain group (GLS ≥ −14%) based on an established clinical reference threshold for global longitudinal strain.

### 2.2 Data collection

Baseline demographic data, pre-existing conditions (including hypertension, diabetes, chronic liver disease, and chronic renal disease), sources of infection, and vital signs were recorded upon admission. Laboratory indicators were collected within 24 h of the diagnosis of sepsis or septic shock. These included C-reactive protein (CRP), white blood cell count (WBC), neutrophil count (NEUT#), lymphocyte count (LY#), platelet count (PLT), lactate (Lac), creatine kinase-MB (CK-MB), myoglobin (MYO), troponin T (TnT-T), N-terminal pro-brain natriuretic peptide (NT-proBNP), procalcitonin (PCT), prothrombin time (PT), activated partial thromboplastin time (APTT), fibrinogen (FIB), D-dimer (D-D), creatinine (Cr), albumin (ALB), globulin (GLB), and total bilirubin (TBIL). The highest (peak) values of TnT-T and NT-proBNP within the initial evaluation window were also recorded. Furthermore, lengths of stay in the intensive care unit (ICU) and hospital, alongside requirements for mechanical ventilation, invasive blood pressure monitoring, and continuous renal replacement therapy (CRRT) were systematically documented.

Echocardiography was performed within 24 h of diagnosis using a Mindray@M9T Color Doppler Ultrasound Diagnostic Instrument equipped with an L12–4s cardiac probe (frame rate: 60–90 frames/second). Patients were instructed to breathe quietly, and routine echocardiography was conducted in the supine or left lateral decubitus position. Measurements of left ventricular diastolic dimension (LVDD), LVEF (using the m-Teich method), lateral wall e-prime (e′), and tricuspid annular plane systolic excursion (TAPSE) were obtained from the parasternal left ventricular long-axis or apical four-chamber views.

Subsequently, a chest lead ECG was connected, and dynamic images of at least three consecutive cardiac cycles were acquired from the apical four-chamber, two-chamber, and three-chamber views. The two-dimensional speckle-tracking imaging mode was activated, and the endocardial reference points of the highest-quality dynamic images were manually traced. The software automatically delineated the endocardial and epicardial boundaries to calculate the apical four-chamber (GLS_A4C), two-chamber (GLS_A2C), three-chamber (GLS_ALAX), and average global longitudinal strain (GLS_AVG). All images were analyzed twice, and the average of the two results was utilized for primary analyses. Ultrasound examinations were performed by trained graduate students under the supervision of the corresponding author, who verified all measurements.

### 2.3 Statistical analysis

Statistical analyses were conducted using SPSS version 26.0 (IBM Corp., Armonk, NY, USA) and R software (version 4.5.1, R Foundation for Statistical Computing, Vienna, Austria). Continuous variables with normal distribution were expressed as mean ± standard deviation (SD) and compared using independent samples *t*-tests. Non-normally distributed continuous variables were presented as medians with interquartile ranges (IQR) and compared using the Mann–Whitney *U*-test. Categorical variables were described using frequencies and percentages, with group comparisons performed via Chi-square or Fisher's exact tests.

To evaluate the intra-observer and inter-observer reproducibility of the speckle-tracking measurements, the Intraclass Correlation Coefficient (ICC) was calculated, and visual agreement was assessed using Bland-Altman plots. The relationships between GLS_AVG and key clinical parameters [Acute Physiology and Chronic Health Evaluation II (APACHE II), SOFA, and myocardial markers] were assessed and visualized using Spearman's rank correlation heatmaps.

Univariate logistic regression analysis was performed to identify potential predictors of 28-day mortality. To rigorously prevent statistical overfitting due to the limited number of outcome events, a restricted multivariate logistic regression model was constructed, incorporating only the most robust predictors (GLS_AVG and SOFA score) to confirm independent prognostic value.

Receiver operating characteristic (ROC) curves were generated to evaluate the predictive performance of the identified risk factors, and the Area Under the Curve (AUC) for GLS_AVG was statistically compared with conventional LVEF using DeLong's test. The optimal cutoff point for GLS_AVG was determined by maximizing the Youden index.

To enhance clinical translation, a predictive nomogram was formulated based on the restricted multivariable model. The internal validity of the nomogram was stringently evaluated through a calibration curve utilizing 1,000 bootstrap resamples. Furthermore, Decision Curve Analysis (DCA) was performed to quantify the clinical net benefit of the predictive models across various threshold probabilities. Finally, exploratory subgroup analyses (presented as forest plots) were conducted to assess the consistency of GLS_AVG across different clinical stratifications. All statistical tests were two-tailed, and a *P*-value < 0.05 was considered statistically significant.

## 3 Results

### 3.1 Baseline characteristics and clinical group comparisons

The study included a total of 46 patients initially diagnosed with sepsis or septic shock. Based on the 28-day prognosis, 35 participants were categorized into the survival group, while 11 were classified into the non-survival group. As detailed in Table 1, there were no significant differences between the two groups regarding baseline demographics (age and sex), underlying comorbidities (such as hypertension and diabetes mellitus), or primary sources of infection (predominantly pulmonary), indicating well-balanced baseline cohorts.

**Table 1:** Baseline characteristics of patients with sepsis or septic shock stratified by 28-day mortality.

Variables	Survival Group (*n* = 35)	Non-survival Group (*n* = 11)	*P* value
Demographics			
Age, years (mean ± SD)	60.51 ± 13.87	54.00 ± 18.56	0.218
Male sex, *n* (%)	20 (57.1)	6 (54.5)	1
Comorbidities, *n* (%)			
Hypertension	16 (45.7)	5 (45.5)	1
Diabetes mellitus	14 (40.0)	3 (27.3)	0.501
Chronic liver disease	5 (14.3)	4 (36.4)	0.186
Chronic renal disease	2 (5.7)	2 (18.2)	0.238
Source of infection, *n* (%)			
Pulmonary	25 (71.4)	9 (81.8)	0.701
Intra-abdominal	2 (5.7)	2 (18.2)	0.238
Urinary tract	8 (22.9)	0 (0.0)	0.169
Bloodstream	2 (5.7)	0 (0.0)	1
Clinical Characteristics			
Septic shock, *n* (%)	14 (40.0)	11 (100.0)	0.002
Mechanical ventilation, *n* (%)	8 (22.9)	9 (81.8)	0.001
CRRT, *n* (%)	11 (31.4)	5 (45.5)	0.625
HR, bpm (mean ± SD)	101.46 ± 21.81	113.36 ± 20.84	0.12
MAP, mmHg (mean ± SD)	84.23 ± 16.14	79.82 ± 26.91	0.616
Length of ICU stay, days [median (IQR)]	5.00 [3.00, 9.00]	5.00 [3.00, 12.50]	0.509
Length of hospital stay, days [median (IQR)]	16.00 [10.50, 21.50]	12.00 [4.50, 14.50]	0.071
APACHE II score (mean ± SD)	15.80 ± 4.05	21.09 ± 5.63	0.001
SOFA score (mean ± SD)	7.63 ± 3.15	11.55 ± 3.11	0.001
Laboratory Findings			
WBC, ×10^9/L [median (IQR)]	11.55 [6.73, 16.02]	6.49 [2.84, 14.26]	0.328
NEUT, ×10^9/L [median (IQR)]	10.19 [6.03, 13.36]	5.78 [2.35, 11.57]	0.321
LY, ×10^9/L [median (IQR)]	0.59 [0.30, 0.79]	0.51 [0.20, 0.70]	0.347
PLT, ×10^9/L [median (IQR)]	151.00 [44.50, 225.00]	125.00 [42.00, 166.00]	0.528
CRP, mg/L [median (IQR)]	158.79 [58.29, 226.11]	171.15 [89.88, 191.94]	0.99
PCT peak, ng/mL [median (IQR)]	10.40 [1.15, 47.35]	52.61 [12.99, 120.82]	0.032
Lactate peak, mmol/L [median (IQR)]	3.70 [1.20, 4.59]	6.40 [2.70, 10.05]	0.022
PT, s (mean ± SD)	15.84 ± 3.92	19.01 ± 4.47	0.029
APTT, s (mean ± SD)	40.47 ± 13.72	46.03 ± 12.04	0.213
FIB, g/L [median (IQR)]	4.67 [3.46, 6.07]	3.43 [2.09, 6.13]	0.268
D-D, mg/L [median (IQR)]	3.86 [2.57, 8.36]	6.14 [2.92, 7.77]	0.528
Cr, μmol/L [median (IQR)]	169.00 [85.00, 260.00]	144.00 [104.00, 343.50]	0.554
ALB, g/L (mean ± SD)	31.95 ± 6.00	25.45 ± 4.72	0.002
GLB, g/L (mean ± SD)	29.25 ± 3.89	27.02 ± 5.36	0.222
TBIL, μmol/L [median (IQR)]	17.40 [11.10, 33.00]	15.10 [11.75, 24.00]	0.738
Cardiac Biomarkers (Peak)			
TnT-T, ng/L [median (IQR)]	0.05 [0.02, 0.14]	0.20 [0.09, 0.32]	0.007
NT-proBNP, pg/mL [median (IQR)]	5,655.00 [2,273.00, 14,476.00]	15,418.00 [13,658.50, 69,998.00]	0.002
CK-MB, U/L [median (IQR)]	1.87 [1.29, 3.16]	3.13 [2.00, 4.92]	0.066
MYO, ng/mL [median (IQR)]	83.10 [53.62, 197.40]	167.00 [77.45, 361.75]	0.315
Echocardiographic Parameters			
LVDD, mm [median (IQR)]	46.00 [41.50, 47.00]	46.00 [40.00, 50.50]	0.99
LVEF, % (mean ± SD)	65.86 ± 9.26	57.82 ± 13.50	0.03
e’, cm/s [median (IQR)]	10.00 [9.00, 12.00]	10.00 [8.25, 10.50]	0.522
TAPSE, mm (mean ± SD)	21.03 ± 3.71	18.36 ± 4.61	0.102
GLS_A4C, % (mean ± SD)	−14.20 ± 3.22	−9.36 ± 4.76	0.008
GLS_A2C, % (mean ± SD)	−15.00 ± 3.82	−10.73 ± 4.10	0.007
GLS_ALAX, % (mean ± SD)	−15.00 ± 3.77	−10.55 ± 4.48	0.009
GLS_AVG, % (mean ± SD)	−14.69 ± 3.13	−10.09 ± 4.18	<0.001

Continuous variables are expressed as mean ± standard deviation (SD) for normally distributed data, or median [interquartile range, IQR] for non-normally distributed data. Categorical variables are presented as frequencies and percentages (*n*, %). Differences between the survival and non-survival groups were evaluated using the independent samples *t*-test, Mann–Whitney *U*-test, or Chi-square/Fisher's exact test, as appropriate. HR, heart rate; MAP, mean arterial pressure; ICU, intensive care unit; APACHE II, Acute Physiology and Chronic Health Evaluation II; SOFA, Sequential Organ Failure Assessment; WBC, white blood cell; NEUT, neutrophil count; LY, lymphocyte count; PLT, platelet count; CRP, C-reactive protein; PCT, procalcitonin; PT, prothrombin time; APTT, activated partial thromboplastin time; FIB, fibrinogen; D-D, D-dimer; Cr, creatinine; ALB, albumin; GLB, globulin; TBIL, total bilirubin; TnT-T, troponin T; NT-proBNP, N-terminal pro-brain natriuretic peptide; CK-MB, creatine kinase-MB; MYO, myoglobin; LVDD, left ventricular diastolic dimension; LVEF, left ventricular ejection fraction; e’, early diastolic mitral annular velocity; TAPSE, tricuspid annular plane systolic excursion; GLS_A4C, apical four-chamber global longitudinal strain; GLS_A2C, apical two-chamber global longitudinal strain; GLS_ALAX, apical long-axis global longitudinal strain; GLS_AVG, average global longitudinal strain.

However, the comparative analysis revealed that non-survivors exhibited a significantly higher incidence of septic shock and mechanical ventilation requirement (*P* < 0.01). Regarding conventional and advanced echocardiographic parameters, both LVEF and GLS_AVG (along with its individual components: GLS_A4C, GLS_A2C, and GLS_ALAX) were significantly worse (depressed) in the non-survival group compared to the survival group.

**Figure 1:**
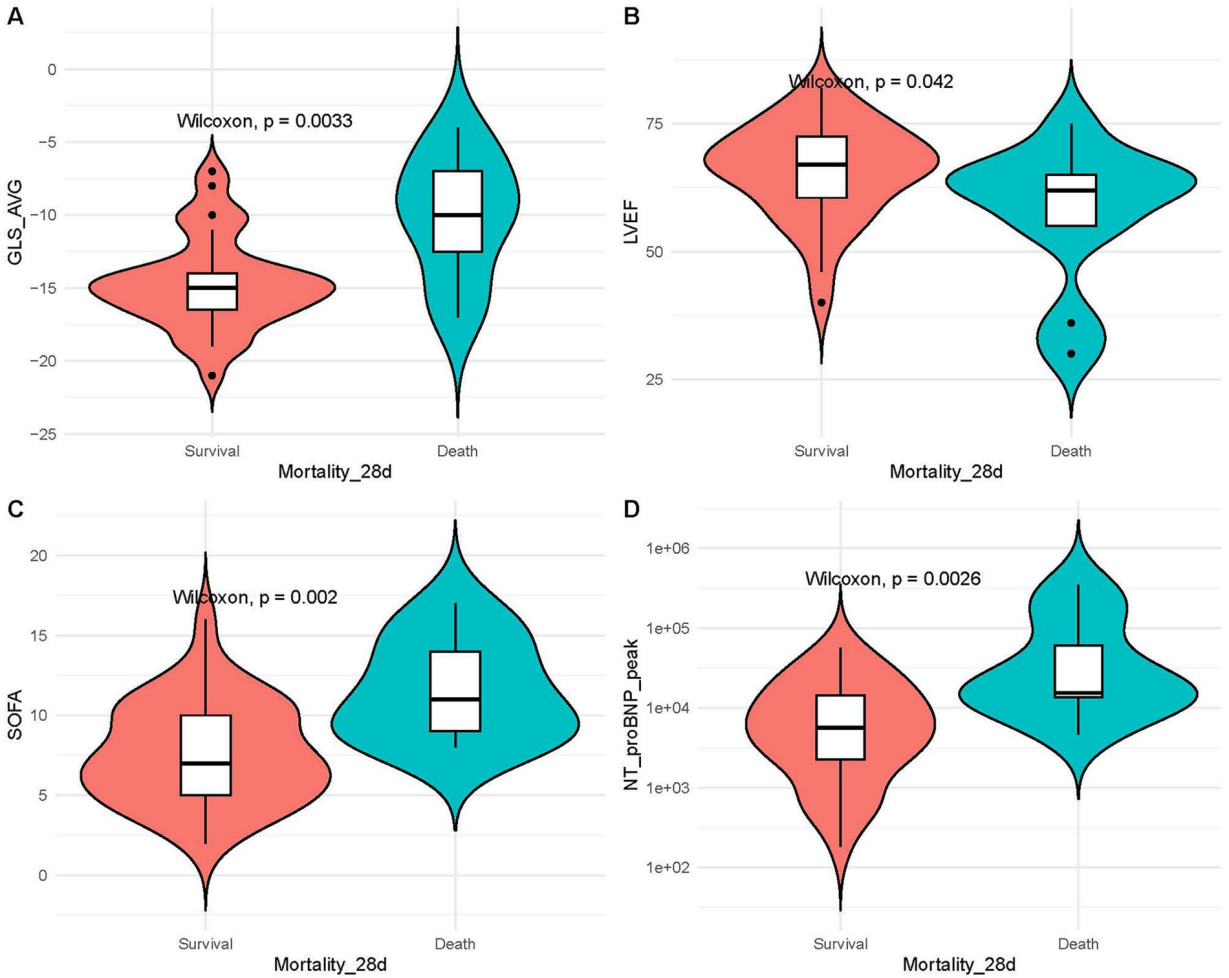
Violin and box plots illustrating the distribution of key clinical and echocardiographic parameters between the survival and non-survival groups. The plots depict the distribution densities of **(A)** GLS_AVG, **(B)** LVEF, **(C)** SOFA score, and **(D)** peak NT-proBNP (log-scaled) in sepsis patients, stratified by 28-day mortality. The internal box plots show the median and interquartile ranges. *P*-values were calculated using the Wilcoxon rank-sum (Mann–Whitney U) test. Non-survivors exhibited significantly worse (less negative) GLS_AVG and higher SOFA scores compared to survivors.

Laboratory indices demonstrated that non-survivors had elevated peak levels of TnT-T and NT-proBNP, accompanied by significantly higher peak procalcitonin (PCT) and lactate levels, as well as reduced albumin (ALB) concentrations (*P* < 0.05). Concurrently, non-survivors presented with higher SOFA and APACHE II scores, indicating a greater severity of multiorgan, metabolic, and myocardial dysfunction. The distribution disparities of key parameters (GLS_AVG, LVEF, SOFA, and NT-proBNP) between the two groups are visually highlighted in the violin plots (Figure 1).

### 3.2 Reproducibility of speckle-tracking echocardiography

**Figure 2:**
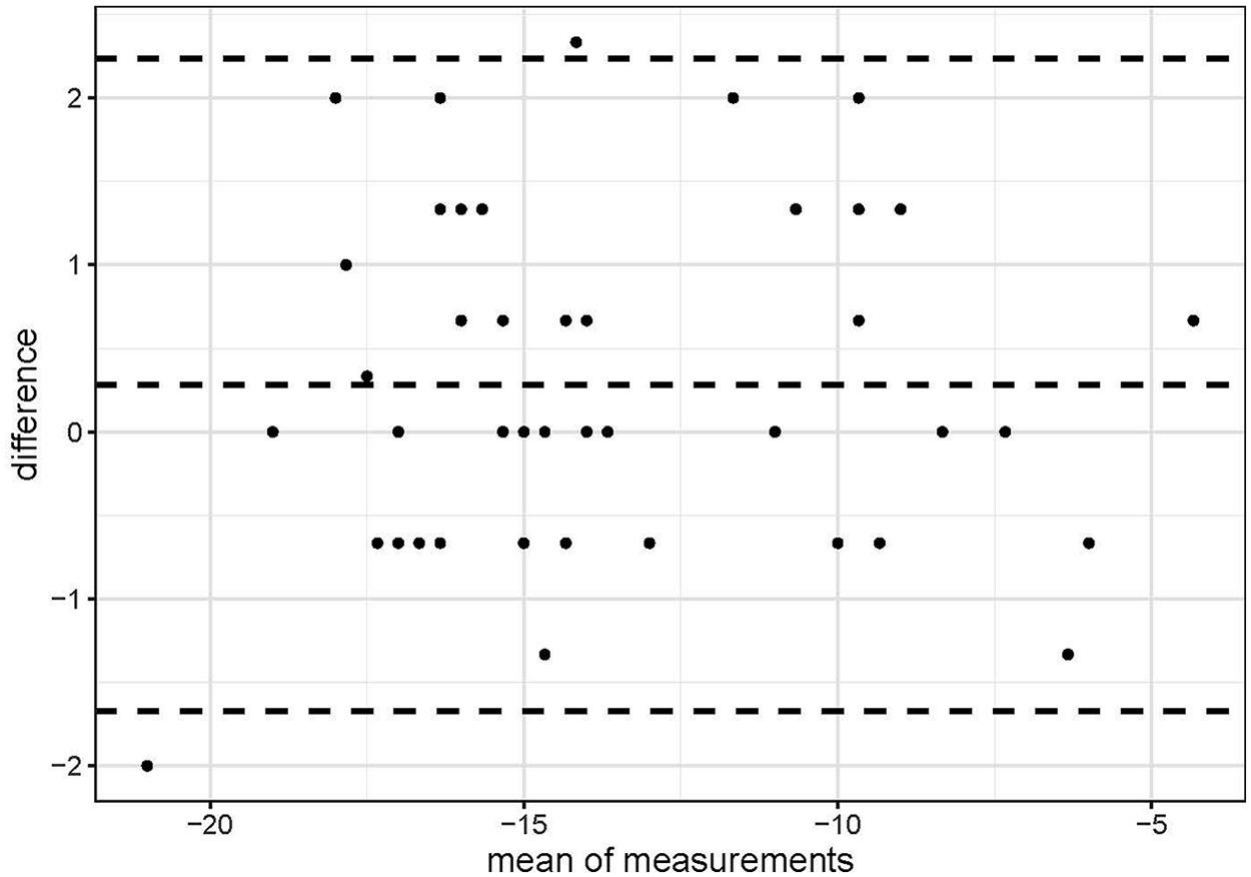
Bland-Altman plot assessing the inter-observer reproducibility of global longitudinal strain (GLS_AVG) measurements. The solid red line represents the mean difference (bias) between the two independent echocardiographic measurements, and the dashed gray lines indicate the 95% limits of agreement (± 1.96 standard deviations). The minimal bias and narrow limits demonstrate the excellent reliability of the speckle-tracking imaging technique.

To validate the reliability of the echocardiographic measurements, inter-observer agreement for GLS_AVG was assessed. The Bland-Altman analysis (Figure 2) demonstrated excellent agreement between the two independent measurements. The mean difference between measurements was minimal (0.28%), with the vast majority of data points falling within the narrow 95% limits of agreement. This indicates that the speckle-tracking technique used in this study is highly reproducible and consistent in an emergency critical care setting.

### 3.3 Correlation analysis of GLS_AVG

**Figure 3:**
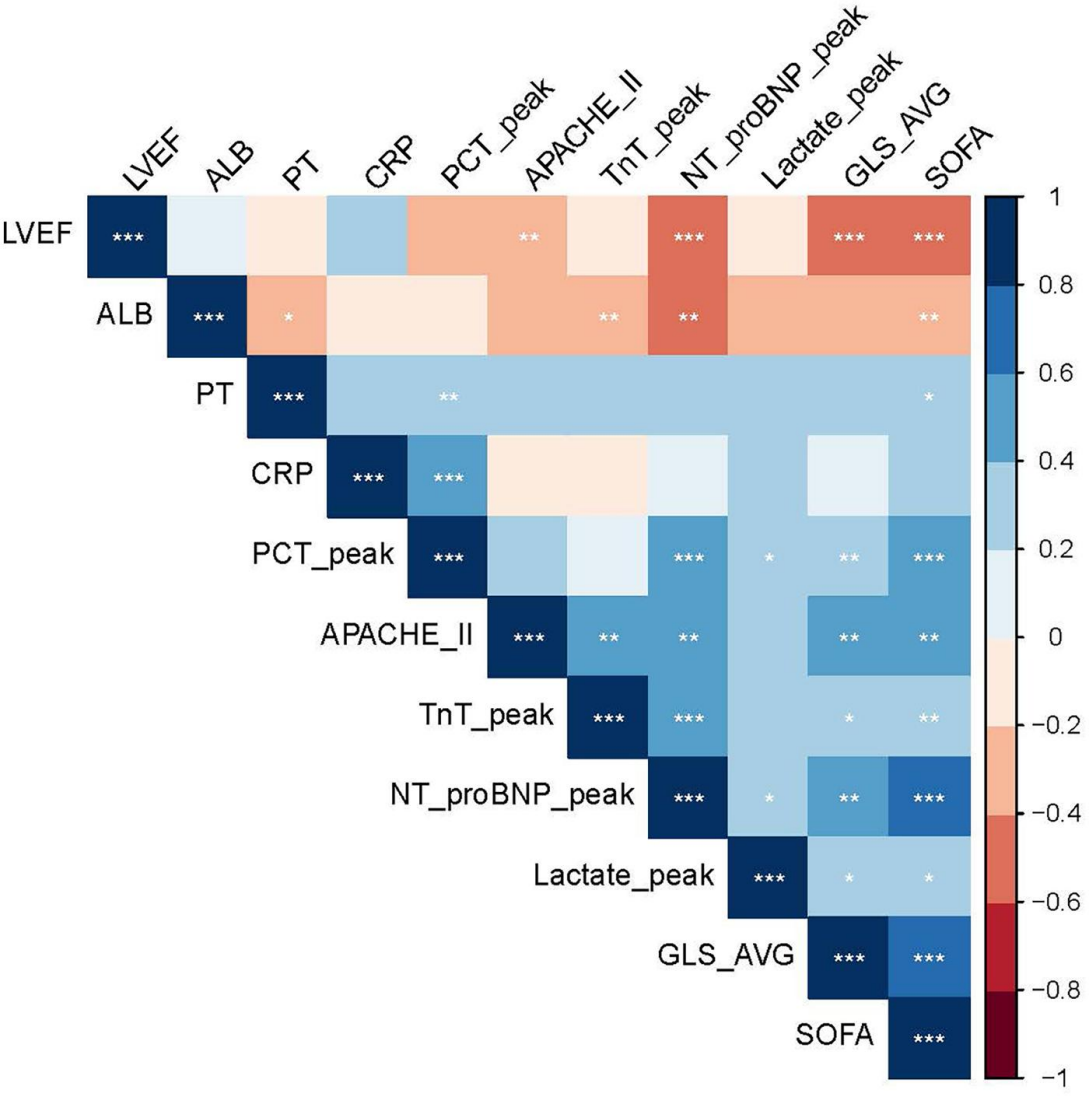
Spearman rank correlation heatmap of GLS_AVG and key clinical variables. Colors represent the direction and magnitude of the Spearman correlation coefficients **(r)**. Blue indicates a positive correlation, while red indicates a negative correlation; darker shades signify stronger correlations. Asterisks denote the level of statistical significance (*P < 0.05, **P < 0.01, ***P < 0.001). GLS_AVG demonstrated significant correlations with critical disease severity scores (SOFA, APACHE II) and myocardial injury biomarkers.

In the analysis of speckle-tracking parameters, GLS_AVG demonstrated significant correlations with several core clinical indices of sepsis severity. As visualized in the Spearman correlation heatmap (Figure 3), GLS_AVG was positively correlated with peak TnT-T (*r* = 0.310, *P* < 0.05), peak NT-proBNP (*r* = 0.424, *P* < 0.01), APACHE II score (*r* = 0.414, *P* < 0.01), and SOFA score (*r* = 0.663, *P* < 0.01). Conversely, GLS_AVG showed a significant negative correlation with LVEF (*r* = −0.501, *P* < 0.01). These robust associations suggest that impaired longitudinal strain is deeply intertwined with systemic inflammation and multiorgan failure.

### 3.4 Prognostic value of GLS_AVG and ROC analysis

Univariate logistic regression analysis identified GLS_AVG, LVEF, SOFA, APACHE II, Lac, ALB, and PT as significant risk factors for 28-day mortality (Table 2). To rigorously avoid overfitting, given the sample size, a restricted multivariate logistic regression model was constructed incorporating only GLS_AVG and the SOFA score. In this model, GLS_AVG maintained a trend toward independent association with 28-day mortality (OR = 1.266, 95% CI: 0.995–1.676, *P* = 0.067), independent of the established SOFA severity score.

**Table 2:** Univariate and restricted multivariate logistic regression analyses for predictors of 28-day mortality.

Variable	OR	CI_lower	CI_upper	P_value
GLS_AVG	1.414134	1.152043	1.833272	0.002787
LVEF	0.93535	0.868989	0.99541	0.04603
SOFA	1.455205	1.152423	1.978699	0.005111
APACHE_II	1.271403	1.08452	1.567051	0.008997
TnT_peak	1.477457	0.621657	3.504264	0.333832
NT_proBNP_peak	1.00004	1.000009	1.00009	0.061105
Lactate_peak	1.257232	1.051388	1.569592	0.022955
ALB	0.798326	0.655163	0.923487	0.00826
PT	1.231904	1.02649	1.554841	0.042899
GLS_AVG (Multi)	1.265675	0.995315	1.675919	0.067055
SOFA (Multi)	1.267121	0.948515	1.762223	0.11725

Variables demonstrating statistical significance in the univariate analysis were considered for the multivariate model. To rigorously prevent statistical overfitting due to the limited number of outcome events, the multivariate logistic regression model was restricted to the two most robust clinical and echocardiographic predictors (GLS_AVG and SOFA score). OR, odds ratio; CI, confidence interval; GLS_AVG, average global longitudinal strain; SOFA, Sequential Organ Failure Assessment; LVEF, left ventricular ejection fraction; APACHE II, Acute Physiology and Chronic Health Evaluation II; ALB, albumin; PT, prothrombin time.

**Figure 4:**
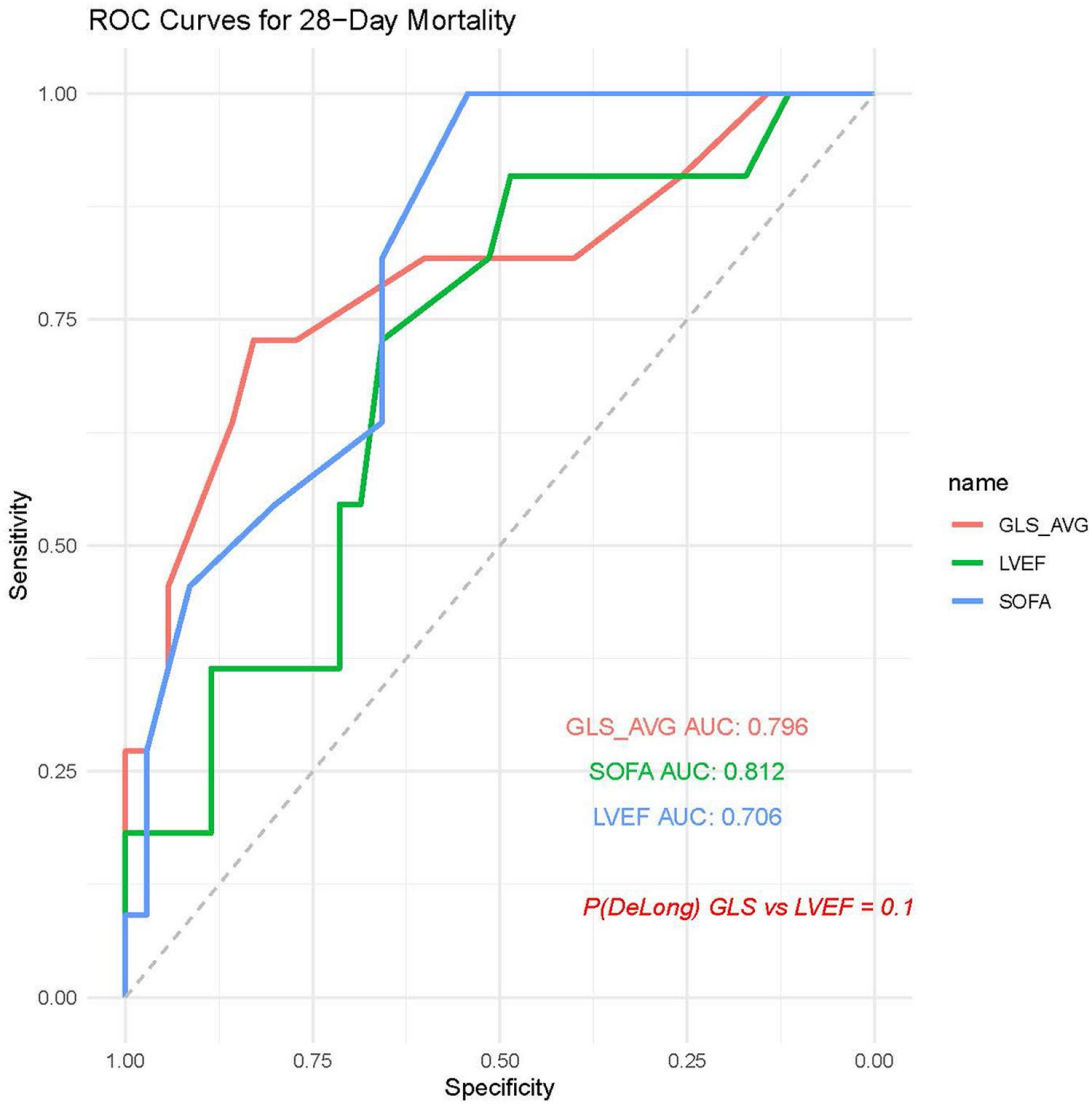
Receiver operating characteristic (ROC) curves for predicting 28-day mortality. The predictive performance of GLS_AVG (red line) is compared against conventional LVEF (blue line) and the clinical SOFA score (green line). GLS_AVG yielded a higher Area Under the Curve (AUC = 0.796) compared to LVEF (AUC = 0.706). DeLong's test was utilized to compare the statistical difference between the ROC curves.

ROC curves were constructed to compare predictive performances (Figure 4). The AUC for GLS_AVG was 0.796. DeLong's test confirmed that GLS_AVG provided an excellent predictive capacity that was superior to conventional LVEF (AUC: 0.706). Utilizing the Youden index, the optimal cutoff value for GLS_AVG to predict 28-day mortality was determined to be >−11.0% (i.e., absolute strain <11.0%), yielding a sensitivity of 72.7% and a specificity of 82.9%.

### 3.5 Clinical predictive model, internal validation, and net benefit

**Figure 5:**
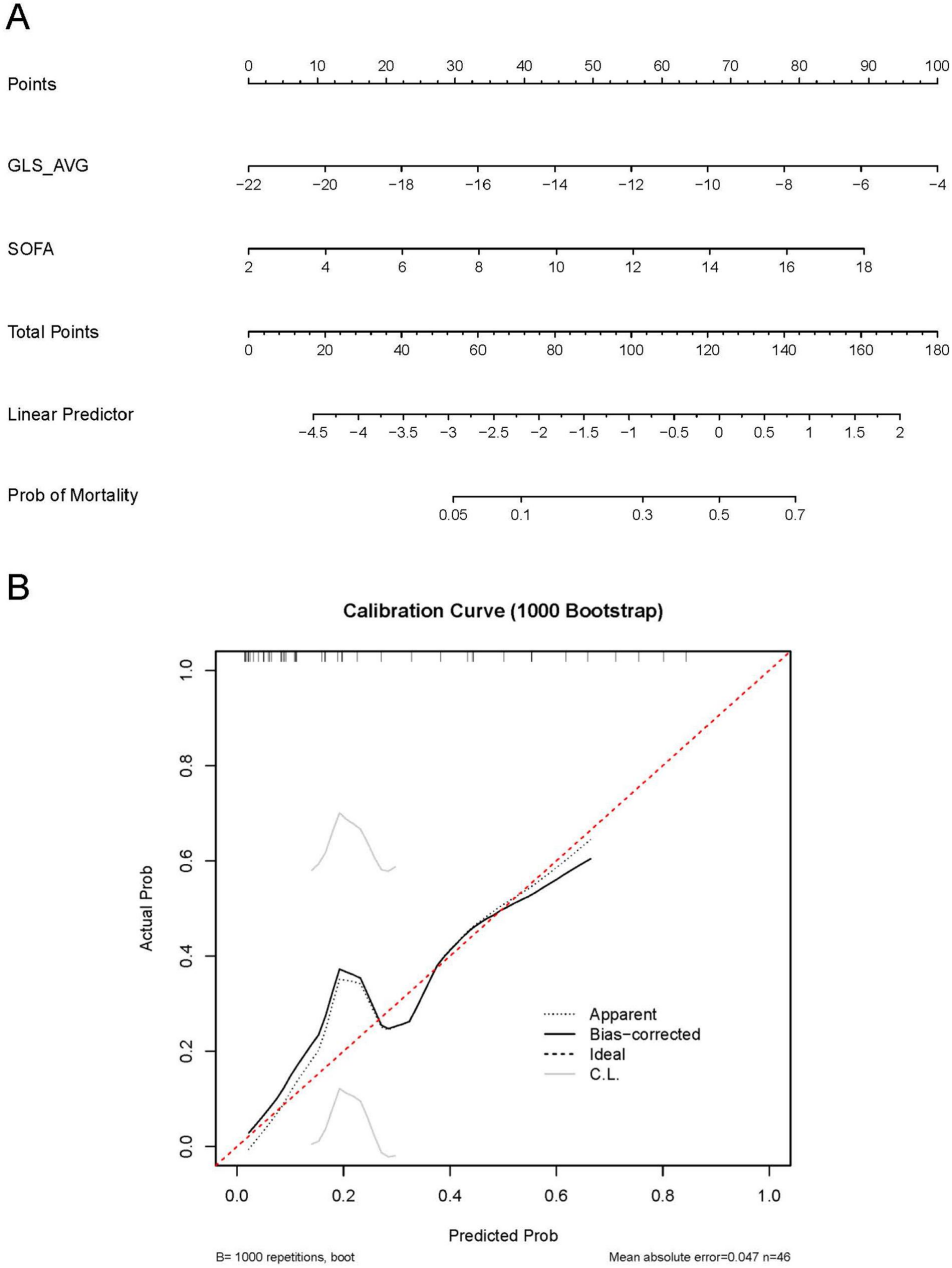
Clinical nomogram and internal calibration curve for predicting 28-day mortality. **(A)** A predictive nomogram developed by integrating GLS_AVG and the SOFA score. Clinicians can obtain the total points by drawing a vertical line from each variable to the “Points” axis, which corresponds to the estimated probability of 28-day mortality on the bottom axis. **(B)** Calibration curve of the nomogram utilizing 1,000 bootstrap resamples. The *x*-axis represents the nomogram-predicted probability, and the *y*-axis represents the actual observed probability. The dashed 45-degree line represents perfect ideal prediction, while the solid line indicates the bias-corrected performance of the model, demonstrating excellent internal consistency.

To individualize risk assessment for emergency and ICU physicians, a nomogram incorporating GLS_AVG and SOFA scores was established to predict the probability of 28-day mortality (Figure 5A). To stringently assess the model's internal robustness, a calibration curve was generated using 1,000 bootstrap resamples (Figure 5B), which demonstrated excellent agreement between the predicted probability and the actual observed mortality.

**Figure 6:**
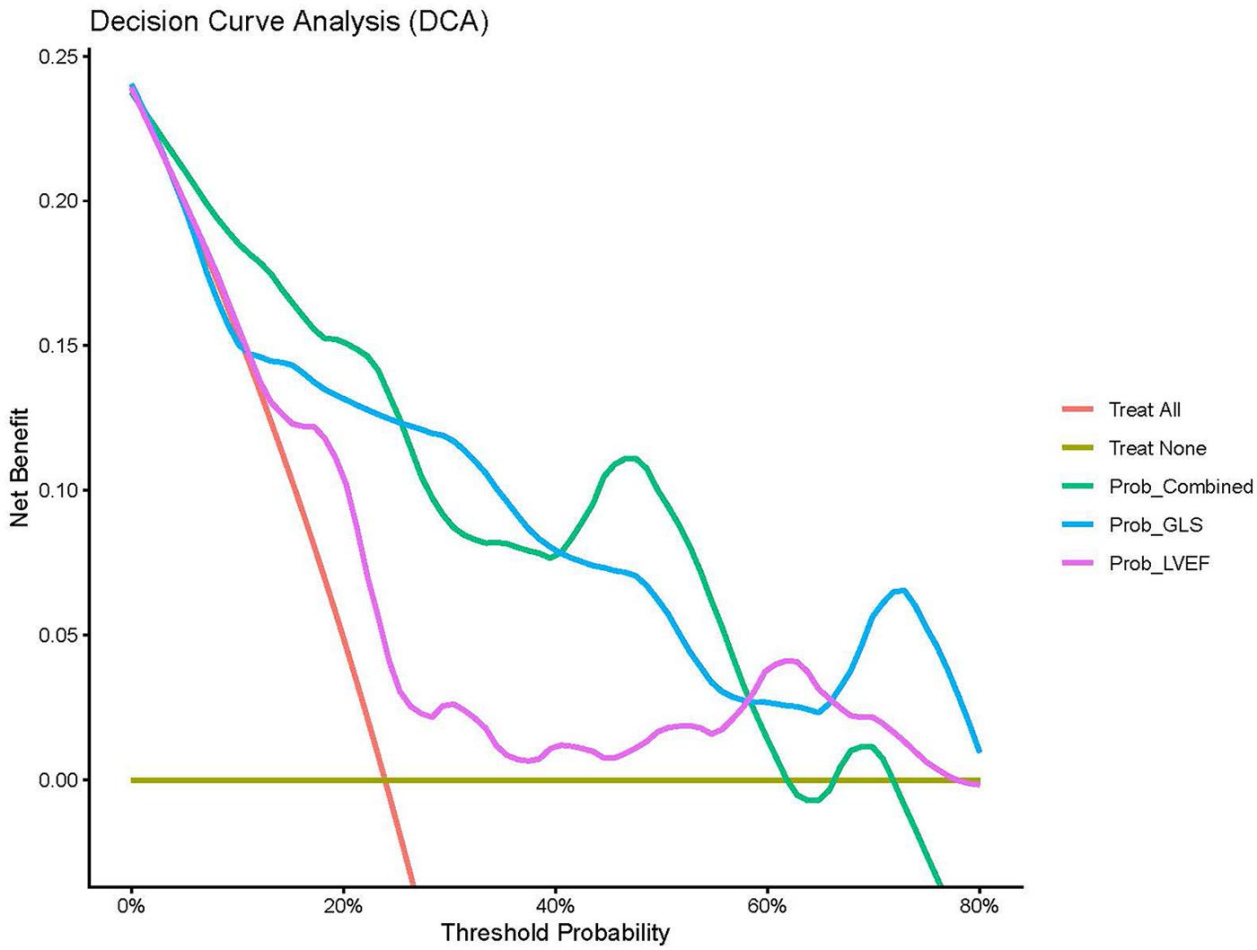
Decision curve analysis (DCA) of the predictive models for 28-day mortality. The DCA evaluates the clinical net benefit of different predictive strategies across a wide range of threshold probabilities. The combined GLS_AVG + SOFA model (Prob_Combined) and GLS_AVG alone demonstrated a consistently higher net benefit compared to relying solely on LVEF, or the extreme clinical strategies of assuming all patients will die (“Treat All”) or no patients will die (“Treat None”). This highlights the tangible clinical utility of incorporating strain imaging into early emergency triage.

Crucially, to quantify the clinical utility of these findings, DCA was performed (Figure 6). The DCA illustrated that utilizing the combined GLS-SOFA model or GLS_AVG alone provided a substantially higher clinical net benefit across a wide range of threshold probabilities compared to relying solely on LVEF, or the extreme “treat-all”/“treat-none” strategies.

### 3.6 Exploratory subgroup analysis

**Figure 7:**
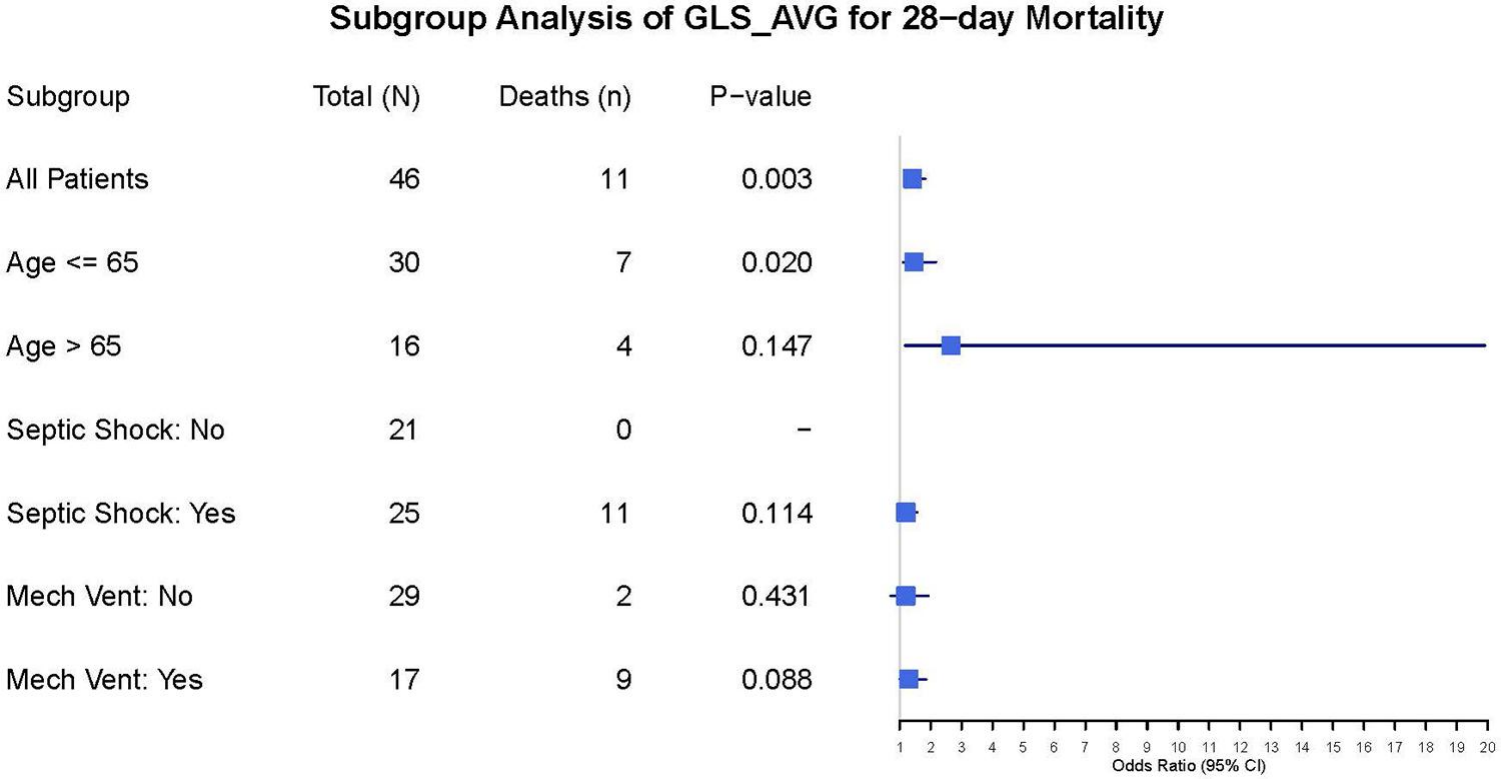
Forest plot of exploratory subgroup analyses for the association between GLS_AVG and 28-day mortality. The plot displays the Odds Ratios (OR) and 95% Confidence Intervals (CI) for GLS_AVG in predicting mortality across various clinical subgroups, including age stratifications, the presence of septic shock, and the requirement for mechanical ventilation. The vertical line at OR = 1 indicates no effect. The directionality of risk remained generally consistent across all subgroups, confirming the broad prognostic applicability of GLS in heterogeneous septic populations.

Exploratory subgroup analyses were conducted to determine the consistency of GLS_AVG as a prognosticator across varying clinical presentations (Figure 7). The forest plot indicated that the increased mortality risk associated with impaired (elevated) GLS_AVG remained directionally consistent across different subgroups, including stratifications by age, the presence of septic shock, and the need for mechanical ventilation. This further supports the broad applicability of GLS_AVG in heterogeneous septic populations.

## 4 Discussion

In this prospective observational study, we evaluated the prognostic utility of early STI in patients presenting to the emergency department (ED) with sepsis or septic shock. Our primary findings indicate that early impairment of GLS_AVG is a powerful, prognostic marker of 28-day mortality, significantly outperforming conventional LVEF. Furthermore, we successfully translated these imaging and clinical findings into a highly applicable, internally validated predictive nomogram. By utilizing DCA, we provided compelling evidence demonstrating the tangible clinical net benefit of incorporating STI-derived GLS into the early triage and hemodynamic management of septic patients.

The early detection of SIMD has historically been hindered by the inherent limitations of conventional echocardiography. While LVEF remains the most universally utilized metric for assessment, our study corroborates previous findings (13) that LVEF severely lacks sensitivity in the early, hyperdynamic phases of sepsis. The complex anatomical arrangement of the ventricular myocardium provides a robust mechanistic explanation for this discrepancy. The left ventricular wall comprises three distinct muscular layers: the superficial oblique fibers, the middle circumferential fibers, and the deep longitudinal subendocardial fibers (14). The subendocardial longitudinal fibers, which govern the base-to-apex shortening of the heart, are subjected to the highest intramural wall stress and are situated at the distal end of the coronary microcirculation. During the early stages of sepsis, the profound systemic inflammatory cascade—driven by the release of cytokines such as tumor necrosis factor-alpha (TNF-α) and interleukin-1 beta (IL-1β)—precipitates microvascular thrombosis, endothelial leakage, and tissue edema (15). These insults disproportionately and preferentially target the vulnerable subendocardium (16).

Consequently, longitudinal deformation (quantified by GLS) is significantly impaired long before the subepicardial and mid-wall circumferential fibers fail. Because LVEF is predominantly driven by the radial thickening of these resilient middle circumferential fibers, the compensatory hyperkinesia of the mid-wall can easily mask the profound functional decline of the subendocardium, resulting in a falsely “normal” or even supranormal LVEF. Our ROC analysis, reinforced by DeLong's test, statistically validated this pathophysiological cascade. We confirmed that GLS possesses a significantly higher diagnostic yield (AUC = 0.796) than LVEF (AUC = 0.706) in identifying life-threatening myocardial depression, echoing the experimental models which demonstrated that strain alterations precede macroscopic LVEF decline (16).

Defining a universal and absolute pathological cutoff for GLS in sepsis remains somewhat controversial due to inter-vendor variability, varying software algorithms, and diverse patient demographics. A comprehensive meta-analysis previously established a normal healthy GLS range between −15.9% and −22.1% (17). In the context of critical illness, however, several studies focusing on severe sepsis have utilized a threshold of −14% to define abnormal longitudinal strain (18). In our ED-based cohort, utilizing the Youden index, we identified an optimal predictive cutoff of −11.0% (i.e., absolute strain < 11.0%) for 28-day mortality. This slightly more depressed diagnostic threshold may reflect the acute, unresuscitated, and highly unstable status of patients at the exact moment of ED triage compared to later ICU assessments. In the ED, profound vasoplegia, extreme tachycardia, and dynamic loading conditions are most pronounced. The fact that GLS maintained its strong predictive value (Sensitivity 72.7%, Specificity 82.9%) despite these chaotic hemodynamic shifts underscores its relatively lower load-dependence compared to traditional ejection fraction measurements.

Furthermore, our correlation analyses elucidate that impaired GLS is not merely an isolated marker of localized cardiac injury but serves as a systemic mirror of multi-organ deterioration. GLS_AVG exhibited robust positive correlations with established severity scoring systems, including SOFA and APACHE II, as well as critical biomarkers such as NT-proBNP and TnT-T. Notably, the concomitant elevation of cardiac markers and creatinine in patients with severe strain abnormalities strongly points toward the early development of secondary organ crosstalk. This interconnected physiological deterioration is emblematic of Cardiorenal Syndrome Type 5, a condition in which a severe systemic insult—such as sepsis—simultaneously damages both the heart and the kidneys (19). Sepsis-induced cardiac dysfunction exacerbates renal injury through a dual mechanism: “forward failure” resulting in inadequate renal arterial perfusion, and “backward failure” causing severe venous congestion and elevated renal interstitial pressure. This vicious pathophysiological cycle perfectly explains why severely impaired GLS was also intricately linked with hyperlactatemia and the necessity for aggressive vasoactive support in our cohort, echoing the findings by Lanspa et al., who correlated poor strain with low central venous oxygen saturation (ScvO2) (20).

The most notable novelty and clinical contribution of the present study lies in bridging the gap between advanced echocardiographic metrics and bedside clinical decision-making. While previous systematic reviews, meta-analyses (21), and experimental models (22, 23) have firmly established the mathematical association between GLS and mortality, translating this parameter into a practical, actionable tool has remained a challenge. To this end, we successfully constructed a predictive nomogram integrating the patient's GLS_AVG and SOFA score. Recognizing the statistical limitations inherent to relatively smaller sample sizes, we stringently validated this predictive model using a 1,000-resample bootstrap calibration technique. The calibration curve demonstrated excellent internal consistency, proving that the model's predictions align closely with actual observed mortality and effectively mitigating the risk of statistical overfitting.

More importantly, traditional metrics like the Area Under the Curve (AUC) only measure diagnostic accuracy without considering the clinical consequences of false-positive or false-negative decisions. To overcome this, our DCA provided compelling evidence that utilizing the combined GLS-SOFA model yields a superior clinical “net benefit” across a remarkably broad spectrum of threshold probabilities. For frontline emergency physicians and intensivists, this implies that utilizing GLS to guide early resuscitative interventions—such as cautious fluid titration, early initiation of inotropes (e.g., dobutamine or levosimendan), or the deployment of mechanical circulatory support—results in demonstrably better patient outcomes compared to relying solely on LVEF or adopting a universal “treat-all” empirical strategy.

Our exploratory subgroup analyses further solidify the universal applicability of GLS. The forest plot demonstrated that the increased mortality risk associated with impaired (elevated) GLS_AVG remained directionally consistent across diverse clinical phenotypes, including stratifications by age (≤65 vs. >65 years), the presence or absence of frank septic shock, and the requirement for invasive mechanical ventilation. The consistency of these findings suggests that the degradation of myocardial mechanics is a fundamental pathway in sepsis pathophysiology, universally detrimental regardless of the patient's baseline demographic or primary source of infection.

Despite these robust findings, this study is subject to several limitations that warrant acknowledgment. Foremost among these is the relatively small sample size (*N* = 46), with only 11 outcome events, which limits the statistical power for definitive multivariable adjustment and constrains the generalizability of our findings. Although we implemented restricted multivariable modeling and rigorous 1,000-resample bootstrap internal validation to mitigate overfitting, these internal validation strategies cannot substitute for external validation. Because it is a single-center prospective study, consequently, the nomogram and cutoff values derived from this cohort should be considered hypothesis-generating and require prospective validation in larger, independent multicenter cohorts before clinical implementation. Although our rigorous internal bootstrap validation and comprehensive DCA provide high confidence in the statistical reliability of our findings, larger multicenter validations are necessary. Second, STI is highly dependent on high-quality acoustic windows. Patients with poor imaging quality, often due to obesity, severe pulmonary edema, or mechanical ventilation challenges, were excluded, which may introduce a degree of selection bias. Third, owing to software limitations on the emergency ultrasound equipment utilized during the study period, right ventricular (RV) longitudinal strain was not systematically evaluated. Given that RV dysfunction is intimately associated with mortality in sepsis and acute respiratory distress syndrome (ARDS) (24), the absence of biventricular strain analysis is a notable limitation. The incremental prognostic value of adding RV strain parameters to our current GLS-SOFA model remains unknown; future prospective studies incorporating serial biventricular strain measurements are needed to determine whether a combined biventricular approach would further improve the nomogram's predictive performance and net benefit on DCA. Until such data emerge, the current nomogram should be applied with awareness that it does not capture the potential contribution of RV dysfunction to mortality risk. Finally, this study captured a single snapshot of myocardial function upon ED admission. Because SIMD is recognized as a dynamic and frequently reversible condition, future studies incorporating serial STI monitoring over the first 72 h of resuscitation would provide invaluable insights into myocardial recovery trajectories.

## 5 Conclusion

In conclusion, STI-derived GLS_AVG provides a highly sensitive, non-invasive, and reliable assessment of subclinical myocardial dysfunction in the crucial early stages of sepsis. By significantly outperforming conventional LVEF and offering demonstrable clinical net benefit through our validated nomogram and decision curve models, GLS represents a vital prognostic tool. The integration of advanced strain imaging into routine emergency and critical care protocols holds immense potential to enhance early risk stratification, guide personalized hemodynamic resuscitation, and ultimately improve survival outcomes in patients grappling with sepsis and septic shock. Nevertheless, these findings should be interpreted as preliminary, given the limited sample size, and warrant validation in larger multicenter studies.

## Data Availability

The original data of this article will be made available by the authors, without undue reservation.
